# Incidence and determinants of medication errors and adverse drug events among hospitalized children in West Ethiopia

**DOI:** 10.1186/s12887-016-0619-5

**Published:** 2016-07-07

**Authors:** Mohammed Gebre Dedefo, Abraham Haileamlak Mitike, Mulugeta Tarekegn Angamo

**Affiliations:** Department of Pharmacy, Wollega University, Nekemte, Oromia Ethiopia; Department of Pharmacy, Jimma University, Jimma, Oromia Ethiopia; Department of Pediatrics, Jimma University, Jimma, Oromia Ethiopia

**Keywords:** Medication errors, Adverse drug events, Children, Nekemte Referral Hospital

## Abstract

**Background:**

Medication errors cause a large number of adverse drug events with negative patient health outcomes and are a major public-health burden contributing to 18.7–56 % of all adverse drug events among hospitalized patients. The aim of this study was to assess the incidence and determinants of medication errors and adverse drug events among hospitalized children.

**Methods:**

A prospective observational study was conducted among hospitalized children in the pediatrics ward of Nekemte Referral Hospital from February 24 to March 28, 2014. Data were collected by using checklist guided observation and review of medication order sheets, medication administration records, and other medical charts of the patients. To identify the independent predictors of medication errors and adverse drug events, backward logistic regression analysis was used. Statistical significance was considered at *p*-value <0.05.

**Results:**

Out of 233 patients who were included in the study, 175 (75.1 %) of patients were exposed to medication errors. From the 1,115 medication orders reviewed, 513 (46.0 %) medication errors, 75 (6.7 %) potential adverse drug events and 17 (1.5 %) actual adverse drug events were identified. Of the 17 adverse drug events, eight (47.0 %) were preventable while nine (53.0 %) were not. Most medication errors were dosing errors (118; 23.0 %), followed by wrong drug (109; 21.2 %) and wrong time of administration (79; 15.4 %). On multivariable logistic regression analysis, length of hospital stay of ≥ 5 days (AOR = 4.2, 95 % CI = 1.7-10.4, *p* = 0.002), and number of medication of 4–6 (AOR = 4.9, 95 % CI = 2.3-10.3, *p* < 0.001) and number of medication of ≥7 (AOR = 10.4, 95 % CI = 3.0-35.9, *p* < 0.001) were independent predictors of medication errors; and length of hospital stay of ≥ 5 days (AOR = 3.5, 95 % CI = 1.2-10.1, *p* = 0.023) and number of disease conditions =2 (AOR = 4.6, 95 % CI = 1.4-15.1, *p* = 0.014) were independent predictors of adverse drug events.

**Conclusion:**

Medication errors and adverse drug events are common on the pediatrics ward of Nekemte Referral Hospital. In particular, children with multiple medications and longer hospital stays, and those with co-morbidities and longer hospital stays, were at greater risk for medication errors and adverse drug events, respectively.

## Background

All adverse drug events (ADEs), adverse drug reactions (ADRs), and medication errors (MEs) fall under the umbrella of medication misadventures. An ADE refers to any injury caused by a medicine [1]. The term ADE includes harm caused by the drug (adverse drug reactions and overdoses) and harm from the use of the drug (including dose reductions and discontinuations of drug therapy). An ADE refers to all ADRs, including allergic or idiosyncratic reactions, as well as MEs that result in harm to a patient. ADRs refer to any unexpected, unintended, undesired, or excessive response to a medicine; it is harm directly caused by the drug at normal doses, during normal use [2]. A ME is any preventable event that has the potential to lead to inappropriate medication use or patient harm; many occur during prescribing, transcribing, dispensing, administering, adherence, or monitoring a drug [1, 2].

If the ME has resulted in actual patient harm, it is categorized as an ADE. An ADE can be either preventable or non-preventable. A potential ADE is a ME that has the potential to harm a patient but which does not actually cause any harm (near-miss) [3]. It is further categorized as intercepted or non-intercepted. An intercepted potential ADE is a drug order that is intercepted before it reaches the patient, whereas a non-intercepted potential ADE has reached the patient but does not cause injury because the patient has sufficient physiological reserves [3].

The top 10 causes of pediatric errors for the 2-year period of the calendar year 1999 to 2000 reported in the United States Pharmacopeia (USP) were the following: performance deficit, procedure or protocol not followed, miscommunication, inaccurate or omitted transcription, improper documentation, drug distribution system error, knowledge deficit, calculation error, computer entry error, and lack of system safeguards [4].

Children are at higher risk for MEs and ADEs for numerous reasons [5]. The weight of a young infant changes rapidly and dosage adjustments may be required as they grow. Frequently there is the lack of an appropriate dosage form for the pediatric patient and adult formulations must be diluted or reformulated for use in children. Absorption, transport, metabolism, and excretion may vary by age [6]. In addition, information or Food and Drug Admiration (FDA) labeling regarding dosing, safety, efficacy, and clinical use in pediatrics is not available or is insufficient, therefore, off-label usage occurs. Without such prescribing, effective therapy would be denied to many children [5, 6]. MEs cause a large number of ADEs with negative patient health outcomes each year and are major public-health burdens representing 18.7–56 % of all ADEs among hospitalized patients [7].

In Ethiopia, a study done in the pediatric ward of Jimma University Specialized Hospital (JUSH) showed that the prevalence of medication administration errors was 89.9 % [8]. Similarly another study from the same setting showed that MEs had an incidence of 55.4 per 100 admissions and ADEs had an incidence of 9.2 per 100 admissions (Chanie et al. 2011, unpublished work).

To the best of our knowledge there are little or no published data on MEs and ADEs among hospitalized children in Ethiopia. This study fills the information gap through assessing the incidence and type of MEs and ADEs; and identifying the factors associated with MEs and ADEs in hospitalized children.

## Methods

### Study setting and period

The research was conducted in the pediatrics ward of Nekemte Referral Hospital, which is found in Nekemte town, West Ethiopia. Nekemte Referral Hospital has clinical divisions such as surgery, gynecology and obstetrics, pediatrics, internal medicine, psychiatry and dermatology. The pediatrics ward contains 47 beds and there are two pediatricians and two general practitioners working in the ward. The study was conducted between February 24 to March 28, 2014.

### Study design

Prospective observational study was conducted among hospitalized children.

### Study population

All children hospitalized on the pediatrics ward of Nekemte Referral Hospital during study period taking at least one medication for treatment or prophylaxis and who stayed for at least 24 h in the ward were included in the study.

### Data collection process and data quality assurance

Data were collected by using different approaches. These included:

- Daily chart review for all admissions until discharge/transfer/death: by visiting the study participants daily and reviewing procedure notes, physician progress notes, pertinent laboratory reports, physician orders, medication administration records, nursing/multidisciplinary progress notes and discharge summary.
- Attending multidisciplinary ward rounds: the principal investigator attended clinical rounds and asked for the presence of any alerts for MEs and ADEs.
- Interview of children and/or parents/caregivers, when further information or clarification of information was required.
- All pediatrics ward staff were informed about the study and invited to take part by submitting voluntary reports of any events in the medication deliver process that they noted during their daily activities.

MEs and ADEs was classified in accordance of standard books like, “Pocket book of pediatric Hospital care: Ethiopia” as a reference [9], for additional information World Health Organization (WHO) pocket book of hospital care for children [10], Lexi-Comp’s Pediatric Dosage Handbook [11] and British National Formulary (BNF) for children [12] were used. In addition, actual occurrences of events for reliability that was originally reviewed by principal investigator, severity and preventability was evaluated by a panel of two health professionals (one pediatrician and one clinical pharmacist), who independently categorized the events using a prepared reviewer form. When disagreement affected classification of an event, the reviewers reach consensus through discussion.

Data were collected by using a checklist which was adapted from a checklist prepared for the California Health Care Foundation for addressing MEs in hospitals [13], during hospitalization by visiting the wards daily and examining all relevant patient records. The pediatric trigger toolkit was used for efficient chart review and identification of adverse drug events [14]. The severity of ADE was reported by using the detailed scale published by the National Coordinating Council for Medication Error Reduction and Prevention (NCC MERP) [14]. The Naranjo ADR probability scale was also used to establish the likelihood of ADR occurrence [15].

### Data analysis and interpretation

After data collection, data were entered into the Statistical Package for the Social Sciences (SPSS) version 16 for analysis. Odds ratio with 95 % confidence interval, along with binary and multiple logistic regression was used to assess the significance and strength of association. All factors with a *p*-value <0.25 in the bivariable logistic regression analysis were further entered into the multivariable model to control confounding effects. In multiple logistic regression a *p*-value <0.05 was used as statistically significant. Rates of MEs and ADEs were reported per 100 orders, 100 admissions, and 100 patient-days [14].

### Ethical considerations

Ethical clearance was obtained from the Ethical Review Committee of Jimma University, College of Public Health and Medical Sciences. This committee wrote a formal letter of permission dated the 12th February 2014; reference number “RPGO/295/2014” to Nekemte Referral Hospital to seek its cooperation and access to the data. Patient’s written informed consent was obtained after explaining about the purpose and procedures of the study. When the child was too young to provide written informed consent, it was obtained from parents/caregivers. In addition all the responses were kept confidential. For those patients in whom serious MEs and ADEs were detected, these were brought to the attention of the appropriate staff immediately together with strategies to manage them.

### Definitions of terms

For the purpose of this study the following definitions were adopted with regards to MEs and ADEs:

- Omission error: The failure to administer an ordered dose to a patient before the next scheduled dose, if any [16].
- Wrong time error: Administration of medication outside a predefined time interval from its scheduled administration time (if there is greater than 1 h difference between the ordered time and the time the medication is administered) [16, 17].
- Wrong dosage-form error: Administration to the patient of a drug product in a different dosage form than ordered by the prescriber [16].
- Deteriorated drug error: Administration of a drug that has expired or for which the physical or chemical dosage-form integrity has been compromised [16].
- Wrong dose error: Administration to the patient of a dose that is greater than or less than the amount ordered by the prescriber or administration of duplicate doses to the patient, i.e., one or more dosage units in addition to those that were ordered [16].
- Non-adherence: Inappropriate patient behavior regarding adherence to a prescribed medication regimen [16].
- Wrong route: Includes order written for wrong route, transcribed for wrong route and medication administered to a patient using a different route than ordered [17].
- Lack of knowledge of the medication: Inadequate knowledge of indications for use, available dosage forms, appropriate doses, routes and compatibilities [18].
- Lack of information about the patient: Nurse, physician or pharmacist was unaware of an important aspect of the patient’s condition [18].
- Preventable: Events where a breach of standard professional behavior or technique was identified, or where necessary precautions were not taken, or where the event was preventable by modification of behavior, technique or care [19].
- Non-Preventable: Events where no obvious breach of standard professional behavior or technique occurred, and where necessary precautions were taken, and where no clearly known alteration in method or care exists to prevent the event. Examples of non-preventable ADEs include the following: Dermatological reactions from unknown allergens; known side effects without identified mitigation strategies; Known side effects that are accepted for the benefit of the drug (i.e. nausea with chemo-therapy) [19].

Severity of MEs and ADEs were reported by using the detailed scale published by the NCC MERP, which is categorized through A to I. Categories A through D of the NCC MERP Index are relevant to MEs and Categories E through I of the NCC MERP Index are relevant to ADEs [14]:

- Category A: Circumstances or events that have the capacity to cause error.
- Category B: An error occurred but the error did not reach the patient.
- Category C: An error occurred that reached the patient but did not cause patient harm.
- Category D: An error occurred that reached the patient and required monitoring or intervention to confirm that it resulted in no harm to the patient and/or required intervention to preclude harm.
- Category E: An error occurred that resulted in the need for treatment or intervention and caused temporary patient harm.
- Category F: An error occurred that resulted in initial or prolonged hospitalization and caused temporary harm.
- Category G: An error occurred that resulted in permanent patient harm.
- Category H: An error occurred that resulted in near-death event (e.g. cardiac arrest).
- Category I: An error occurred that resulted in patient death.

## Results

The study included a total of 233 patients, cumulative hospital stay of 999 patient-days, and 1,115 medication orders. More than half of the patients, 149 (63.9 %) were males. Thirty six (15.5 %) of the cases were neonates, 75 (32.2 %) infants, 43 (18.5 %) toddlers, 31 (13.3 %) preschoolers, 29 (12.4 %) school-aged children, and 19 (8.2 %) adolescents. Among 233 patients, 162 (69.5 %) stayed in hospital for < 5 days and 71 (30.5 %) stayed for ≥ 5 days. More than half of the patients, 139 (59.7 %) had a single diagnosed disease. Of the patients, more than half 128 (54.9 %) took 4–6 medications during hospitalization. Intravenous alone was the most frequently used route of administration as shown in Table 1.

**Table 1:** Patient profiles and Medication related factors among hospitalized children in Nekemte Referral Hospital, West Ethiopia, from February 24 to March 28, 2014 (*n* = 233)

Variables	Categories	Frequency	Percent
Sex	Female	84	36.1 %
	Male	149	63.9 %
Age Category in days	≤28 (Neonates)	36	15.5 %
	29-365 (Infants)	75	32.2 %
	366-1095 (Toddlers)	43	18.5 %
	1096-1825 (Preschoolers)	31	13.3 %
	1826-3650 (School-aged children)	29	12.4 %
	3651-5110 (Adolescents)	19	8.2 %
Length of hospital stay in days	<5	162	69.5 %
	≥5	71	30.5 %
Number of disease	1	139	59.7 %
	2	79	33.9 %
	≥3	15	6.4 %
Number of medication used per patient	1-3	63	27.0 %
	4-6	128	54.9 %
	≥7	42	18.0 %
Route of administration	PO	42	18.0 %
	IV	107	45.9 %
	IV + PO	72	30.9 %
	Others	12	5.2 %

*PO* Per Oral

*IV* Intravenous

The most common cause of MEs and ADEs was lack of knowledge/information about the medication accounting for 144 (61.8 %) of all cases (Table 2), which was evidenced by inadequate knowledge of indications about medication use, available dosage forms, appropriate dosing, appropriate routes for administration and drug compatibility in 53 (22.7 %), 3 (1.3 %), 83 (35.6 %), 8 (3.4 %) and 52 (22.3 %) cases, respectively. By drug class, 167 (71.7 %) patients encountered MEs and ADEs due to antimicrobials, 44 (18.9 %) patients due to electrolytes and fluids, 23 (9.9 %) patients due to analgesics with the remainder due to other class of drugs.

**Table 2:** Health professional related factors related to MEs and ADEs among hospitalized children in Nekemte Referral Hospital, West Ethiopia, from February 24 to March 28, 2014 (*n* = 233)

Variables	Frequency	Percent
Lack of information about the patient	10	4.3 %
Lack of knowledge of the medication	144	61.8 %
Incomplete medication order processed	22	9.4 %
Patient identification not checked	1	0.4 %
Illegible physician handwriting	9	3.9 %

The incidence of MEs per 100 orders, per 100 admissions and per 100 patient days was 46.0, 220.2 and 51.4, respectively. The incidence of potential ADEs per 100 orders, per 100 admissions, and per 100 patient days was 6.7, 32.2 and 7.5, respectively. The incidence of ADEs per 100 orders, per 100 admissions and per 100 patient days was 1.5, 7.3 and 1.7, respectively (Table 3).

**Table 3:** Incidence of MEs and ADEs among hospitalized children in Nekemte Referral Hospital, West Ethiopia, from February 24 to March 28, 2014 (*n* = 233)

Variables	Total	Number per 100 orders	Number per 100 admissions	Number per 100 patient days
Medication orders	1115	NA	478.5	111.6
Medication errors	513	46.0	220.2	51.4
Potential ADEs	75	6.7	32.2	7.5
ADEs	17	1.5	7.3	1.7
ADRs	9	0.8	3.8	0.9

*NA* Not applicable

Out of 233 patients, 175 (75.1 %) experienced at least one error, whilst 93 (39.9 %) experienced 3 or more errors. Overall, there were 513 MEs from 1115 medication orders over the 999 patient days. There were 75 (32.2 %) potential ADEs, of which 15 (20 %) were intercepted while 60 (80 %) were not. Of the 175 (75.1 %) patients who encountered MEs 8 (4.6 %) patients developed ADEs. Overall, 17 (7.3 %) ADEs were identified, of which 8 (47 %) were preventable while 9 (53 %) were not. Out of the 17 ADEs 9 were ADRs, according to the Naranjo algorithm score, 1 (11 %) ADRs classified as “possible ADRs” with a probability score of 1 to 4, and 8 (89 %) events were defined as probable with a probability score of 5 to 8. There is no ADR which is classified as definite ADR (Table 4). Of the ADEs that occurred 13 (76.5 %) necessitated discontinuation of the drug while 4 (23.5 %) necessitated medication dosage change. All of the patients with ADRs recovered without sequelae (Table 4).

**Table 4:** MEs, Potential ADEs and ADEs among hospitalized children in Nekemte Referral Hospital, West Ethiopia, from February 24 to March 28, 2014 (*n* = 233)

Variables	Categories	Frequency	Percent
Patients with a ME	No	58	24.9 %
	Yes	175	75.1 %
Number of MEs per patient	0	58	24.9 %
	1	35	15.0 %
	2	47	20.2 %
	≥3	93	39.9 %
Patient with a potential ADE	No	158	67.8 %
	Yes	75	32.2 %
Potential ADE intercepted	No	60	80 %
	Yes	15	20 %
ADE occurred	No	216	92.7 %
	Yes	17	7.3 %
ADR occurred	No	224	96.1 %
	Definite	0	-
	Probable	8	3.4 %
	Possible	1	0.4 %
Category of ADE	Preventable	8	47 %
	Non-preventable	9	53 %

The most common MEs were dosing errors (118; 23 %), followed by wrong drug (109; 21.2 %), wrong time (79; 15.4 %) and deteriorated drug (75; 14.6 %). The most common stage at which MEs occurred was physician ordering 235 (45.8 %) (Table 5). Of the errors that happened during physician ordering, 20 (8.5 %) were intercepted before reaching the patient, of these 15 (75 %) were intercepted during transcription, while 5 (25 %) were intercepted during dispensing. Of the errors that happened during transcription, 8 (66.7 %) were intercepted before reaching the patient, of these 4 (50 %) were intercepted during dispensing, while 4 (50 %) were intercepted during administration. Of the errors that happened during dispensing, 15 (71.4 %) were intercepted before reaching the patient during administration (Table 5).

**Table 5:** Type of MEs and stages at which error occurred among hospitalized children in Nekemte Referral Hospital, West Ethiopia, from February 24 to March 28, 2014 (*n* = 233)

Variables		Frequency	Percent
Type of medication error	Wrong drug	109	21.2 %
	Wrong patient	1	0.2 %
	Wrong dose	118	23 %
	Wrong dosing schedule	9	1.8 %
	Wrong route	12	2.3 %
	Wrong time	79	15.4 %
	Deteriorated drug	75	14.6 %
	Omission	21	4.1 %
	Wrong dosage form	6	1.2 %
	Non-adherence	23	4.5 %
	Monitoring error	43	8.4 %
	Other medication errors^a^	17	3.3 %
Stage of error	Physician ordering	235	45.8 %
	Transcribing	12	2.3 %
	Dispensing pharmacist	21	4.1 %
	Nurse administering	179	34.9 %
	Patient monitoring	43	8.4 %
	Other^b^	23	4.5 %

^a^ Medication errors that does not fall into one of above categories

^b^Medication errors that happen due to patient non-adherence

Regarding severity of the MEs, of the errors encountered in the study using the more detailed scale published by the NCC MERP, 7 (3 %) were classified as Category A (circumstances or events that have the capacity to cause error), 48 (20.6 %) were classified as Category B (an error occurred but the error did not reach the patient) and 147 (63.1 %) were classified as Category C (an error occurred that reached the patient but did not cause patient harm).

Regarding severity of the ADEs, of the 17 ADEs 8 (47.1 %) were associated with error and using the more detailed scale published by the NCC MERP, 7 (41.2 %) were classified as Category E (an error occurred that resulted in the need for treatment or intervention and caused temporary patient harm) and 1 (5.9 %) were classified as Category F (an error occurred that resulted in initial or prolonged hospitalization and caused temporary harm). None was associated with permanent harm, or death.

The bivariable analysis showed that MEs were associated with length of hospital stay, number of disease conditions, number of medications and route of administration. However, other factors did not show statistically significant association with MEs (Table 6).

**Table 6:** Bivariable and Multivariable analysis of factors associated with MEs among hospitalized children in Nekemte Referral Hospital, Western Ethiopia, from February 24 to March 28, 2014

Variables	Categories	COR (95 % CI)	*P* value	AOR (95 % CI)	*P* value
Length of hospital stay in days	<5	1.00		1.00	
	≥5	4.20(1.80-9.81)	*P* = 0.001	4.24(1.73-10.40)	*P* = 0.002
Number of disease	1	1.00			
	2	2.50(1.23-5.10)	*P* = 0.012	---	
	≥3	1.79(0.48-6.68)	*P* = 0.385	---	
Number of medication used per patient	1-3	1.00		1.00	
	4-6	4.974(2.53-9.76)	*P* = 0.000	4.90(2.33-10.32)	*P* = 0.000
	≥7	9.806(3.13-30.74)	*P* = 0.000	10.39(3.01-35.92)	*P* = 0.000
Route of administration	PO	1.00		1.00	
	IV	3.36(1.51-7.47)	*P* = 0.003	2.12(0.87-5.15)	*P* = 0.099
	IV + PO	2.20(0.97-5.00)	*P* = 0.060	0.93(0.35-2.45)	*P* = 0.877
	Others	0.68(0.19-2.47)	*P* = 0.557	0.54(0.12-2.40)	*P* = 0.416

*COR* Crude odds ratio

*AOR* Adjusted odds ratio

Factors that were significantly associated with MEs at *p* < 0.05 with multivariable analysis were length of hospital stay and number of medication used per patient. While dealing with these factors, patients who stayed in the hospital for ≥ 5 days are almost 4 times more likely to have MEs than patients who stayed in the hospital for < 5 days (AOR = 4.2, 95 % CI = 1.7-10.4, *p* = 0.002). Patients who have used 4–6 medications are almost 5 times more likely to have MEs than patients who have used 1–3 medications (AOR = 4.9, 95 % CI = 2.3-10.3, *p* < 0.001), similarly patients who used ≥7 medications are almost 10 times more likely to have MEs than patients who used 1–3 medications (AOR = 10.4, 95 % CI = 3.0-35.9, *p* < 0.001) (Table 6).

The bivariable analysis showed that ADEs were associated with length of hospital stay and number of disease conditions. However, other factors did not show a statistically significant association with ADEs (Table 7).

**Table 7:** Bivariable and multivariable analysis of factors associated with ADEs among hospitalized children in Nekemte Referral Hospital, Western Ethiopia, from February 24 to March 28, 2014

Variables	Categories	COR (95 % CI)	*P* value	AOR (95 % CI)	*P* value
Length of hospital stay in days	<5	1.00		1.00	
	≥5	4.77(1.69-13.46)	*P* = 0.003	3.46(1.18-10.13)	*P* = 0.023
Number of disease	1	1.00		1.00	
	2	6.05(1.88-19.45)	*P* = 0.003	4.55(1.37-15.12)	*P* = 0.014
	≥3	2.41(0.25-23.09)	*P* = 0.445	2.06(0.21-20.31)	*P* = 0.537

*COR* Crude odds ratio

*AOR* Adjusted odds ratio

Factors that were significantly associated with ADEs using multivariable analysis were length of hospital stay and number of diseases. While dealing with these factors, patients who stayed in the hospital for ≥ 5 days are 3.5 times more likely to have ADEs than patients who stayed for < 5 days (AOR = 3.5, 95 % CI = 1.2-10.1, *p* = 0.023). Patients who diagnosed with two concomitant diseases are 4.6 times more likely to have ADEs than patients who diagnosed with one disease (AOR = 4.6, 95 % CI = 1.4-15.1, *p* = 0.014) (Table 7).

## Discussions

In this prospective study, we found that 75.1 % of patients had been exposed to at least one ME and 39.9 % of patients had 3 or more errors. Different strategies should be used to decrease the high rate of medication errors; development and implementation of a computerized physician order entry (CPOE) system, ward-based clinical pharmacists, avoidance of verbal orders, verbal orders read back, medication reconciliation, double check and patient (parent) active participation in care [20]. Nearly comparable results were reported from Jimma University Specialized Hospital Study, where 55.4 % of the patients experienced at least one medication error (Chanie et al. 2011, unpublished work). However, this result is nearly triple that of the study conducted in USA, where 28.6 % of patients had at least one ME and 5.7 % patients had 3 or more errors [5]. This difference might be due to the differences in the hospital settings such as differences in training levels of health care professionals, availability of support system and composition of health care team and differences in data collection methods, differences in the definitions of terms and interpretations of the errors.

Pediatrics pose a unique set of risks of medication errors, predominantly because of the need for dosage calculations, which are individually based on the patient’s weight, age or body surface area, and their condition. This increases the likelihood of errors, particularly dosing errors. For potent drugs, when only a small fraction of the adult dose is required for children, it becomes very easy to cause 10-fold or greater dosing errors because of miscalculation or misplacement of the decimal point [21]. The present study finding showed that the most frequent MEs were dosing errors 23 %, which is comparable with the results of USA studies, 28 % and 28.4 % [5, 22]. Similar results, 29.1 % were reported regarding dosing errors from Jimma University Specialized Hospital Study (Chanie et al. 2011, unpublished work).

The medication process is significantly prone to errors, especially during prescription and drug administration. Implementation of medication error reduction strategies is required in order to increase the safety and quality of pediatric healthcare [23]. In this study, the most common stage for MEs was physician ordering 45.8 %. However, this finding is lower than the studies conducted in USA in which physician ordering was found to be the most common stage for errors, contributing to 74 % [5] and 77.8 % [22] of errors. These differences are probably due to errors like no or wrong date on prescription paper, missing or wrong weight were included in their study. However, this finding is not similar with another study in USA [4] and Jimma University Specialized Hospital Study (Chanie et al. 2011, unpublished work) that reported administration errors accounting for 51 % and 54.3 % of detected errors, respectively was the most common stage of errors. The difference might be due to differences in the types of medication errors that the study included.

In this study, the length of hospital stay was significantly associated (*P* = 0.001) with MEs and it was one of the independent predictors of MEs. The present finding is consistent with that reported by other studies from Jimma University Specialized Hospital (Chanie et al. 2011, unpublished work) and USA [24, 25]. This is because as the patient hospital stay is prolonged the patient will be exposed to a number of administrations of medication and this may lead to the occurrence of MEs.

As the number of disease conditions increased there could be increased medication prescription that is required for the treatment of the disease conditions and this may lead to the occurrence of MEs. The present study showed that the number of diseases a child had was significantly associated (*P* = 0.012) with MEs in bivariable analysis, but generally number of diseases was not independent predictor of MEs in multivariable analysis (*P* > 0.05). However, a study done in USA by Ahuja et al. showed that there was a statistically significant association between the number of disease conditions and MEs in multivariable analysis [25].

The more medications a patient is consuming, the more likely for the occurrence of MEs. The present study showed that the number of medications used by the patient was significantly associated (*P* < 0.001) with MEs and it was one of the independent predictors of MEs. This is in-line with the study from Jimma University Specialized Hospital (Chanie et al. 2011, unpublished work).

In this study, the route of administration had a significantly associated difference for intravenous administration (*P* = 0.003) with oral route of administration, which was considered as reference in bivariable analysis, but generally route of administration was not shown to be a predictor of MEs in multivariable analysis (*P* > 0.05). The present finding is consistent with the report from Jimma University Specialized Hospital Study [8], USA [5] and Saudi Arabia [26]. However, it is not consistent with a study done in Dessie Hospital which reported that the intravenous route was less likely to be associated with prescribing errors [27]. The probable reason is that the study done in Dessie Hospital studied only prescription errors.

Pediatric patients are vulnerable to ADEs because drugs are less likely have been studied extensively in these age groups, and drug absorption and metabolism are more variable and less predictable in these groups. In this prospective study, we found that 7.3 % of patients who were seen in pediatrics ward have been exposed to ADEs; of these 47 % were preventable, while 53 % were not. The result of this study is comparable to a study done in Jimma University Specialized Hospital (Chanie et al. 2011, unpublished work) in which 9.2 % of patients were subjected to ADEs, with 32.7 % classified as preventable and a study in China [28] which identified 6 % of patients were exposed to ADEs of which 61 % classified as preventable. However, the results are higher than a study done in USA which identified 2.3 % of patients suffered ADEs of which 19 % were classified as preventable [5]. The possible reasons for the difference might be due to differences in the methods of detection as this study used the pediatrics trigger toolkit to efficiently identify ADEs.

Regarding the ADRs this study found a higher incidence of ADRs (3.8 per 100 admissions) than the studies done in Italy, 0.9 % [29] and France, 2.64 % [30]; and a lower incidence than the studies done in Germany, 31.8 % [31], Saudi Arabia, 8.2 % [32] and Brazil, 12.5 % [33]. The possible reasons for the differences might be due to differences in the length of hospital stay of patients and also methods used to detect events. The present study was done on a small number of patients and short study duration which could be the additional reasons.

In this study, the length of hospital stay was significantly associated with ADEs and it was one of the independent predictors of ADEs. The present finding is consistent with that reported by other studies from China [28], Jimma University Specialized Hospital Study (Chanie et al. 2011, unpublished work) and Brazil [33].

Multiple diseases make patients more vulnerable to ADEs due to the presence of many diseases and the use of many drugs. The finding of this specific research showed that patients who diagnosed with two diseases are almost 4 times more likely to have ADEs than patients who diagnosed with one disease. This finding is similar to a prospective study done in china [28].

Regarding the number of medications used per patient, the more the medications that are prescribed for a particular patient the greater the risk of ADEs. Studies from China [28] and Brazil [34] have shown that the number of medications used by the patient is significantly associated with ADEs; but this study showed no significant deference between taking higher and lower number of medications. This may be due to the short duration of study period and patients’ short length of hospital stay as indicated in the result which might underestimate detection of ADEs. The present finding is similar to a study done in Jimma University Specialized Hospital (Chanie et al. 2011, unpublished work).

This study has some strengths and limitations. The strength of this study was being a prospective observational study, in which patients were followed from the date of admission to the date of discharge/transfer/death. We used a multidisciplinary approach that examined all aspects of the medication delivery process, from the physician’s order through administration of the drug to the patient and clinical progress.

Despite a comprehensive multidisciplinary approach to data collection, we probably failed to detect some errors, particularly administration errors detected more reliably by trained observers following nurses during routine patient care activities [5]. Because nurses and physicians on the study wards were aware of the study, the Hawthorne effect could have affected both the occurrence and detection of errors. The incidence of errors could have been reduced as the study progressed because we were obliged to take corrective action when we identified serious practice problems. Other limitations were that only two health professionals were involved for assessing severity and preventability which might affect the classification of the event; it is a single ward, single hospital study and therefore might not be generalized to other hospitals in Ethiopia. Small study size and short duration of study were also limitations of this study.

## Conclusions

Generally, the results of this study suggest that MEs, potential ADEs and actual ADEs are common in the Nekemte Referral Hospital’s pediatrics ward. Multivariable logistic regression analysis outputs showed that length of hospital stay and the number of medication the patient took were independent predictors of medication errors. Similarly, length of hospital stay and number of disease conditions were independent predictors of ADEs. Hence, every error should be examined to determine what elements in the medication delivery process allowed it to happen. In this way, those who manage health systems can learn from error and determine what corrections are needed to prevent similar errors in the future.

## Abbreviations

ADE, adverse drug event; ADR, adverse drug reaction; FDA, food and drug administration; JUSH, jimma university specialized hospital; ME, Medication Error; NCC MERP, national coordinating council for medication error reduction and prevention; RPGO, research and postgraduate office; USA, United States of America; WHO, World Health organization
